# Building Radiomics Models Based on Triple-Phase CT Images Combining Clinical Features for Discriminating the Risk Rating in Gastrointestinal Stromal Tumors

**DOI:** 10.3389/fonc.2021.737302

**Published:** 2021-12-07

**Authors:** Meihua Shao, Zhongfeng Niu, Linyang He, Zhaoxing Fang, Jie He, Zongyu Xie, Guohua Cheng, Jian Wang

**Affiliations:** ^1^ Department of Radiology, Tongde Hospital of Zhejiang Province, Hangzhou, China; ^2^ Department of Radiology, Sir Run Run Shaw Hospital, Zhejiang University School of Medicine, Hangzhou, China; ^3^ Hangzhou Jianpei Technology Company, Hangzhou, China; ^4^ Department of Radiology, The First Affiliated Hospital of Bengbu Medical College, Bengbu, China

**Keywords:** gastrointestinal stromal tumors, radiomics models, risk rating, triple-phase CT images, abdomen

## Abstract

We aimed to build radiomics models based on triple-phase CT images combining clinical features to predict the risk rating of gastrointestinal stromal tumors (GISTs). A total of 231 patients with pathologically diagnosed GISTs from July 2012 to July 2020 were categorized into a training data set (82 patients with high risk, 80 patients with low risk) and a validation data set (35 patients with high risk, 34 patients with low risk) with a ratio of 7:3. Four diagnostic models were constructed by assessing 20 clinical characteristics and 18 radiomic features that were extracted from a lesion mask based on triple-phase CT images. The receiver operating characteristic (ROC) curves were applied to calculate the diagnostic performance of these models, and ROC curves of these models were compared using Delong test in different data sets. The results of ROC analyses showed that areas under ROC curves (AUC) of model 4 [Clinic + CT value of unenhanced (CTU) + CT value of arterial phase (CTA) + value of venous phase (CTV)], model 1 (Clinic + CTU), model 2 (Clinic + CTA), and model 3 (Clinic + CTV) were 0.925, 0.894, 0.909, and 0.914 in the training set and 0.897, 0.866, 0,892, and 0.892 in the validation set, respectively. Model 4, model 1, model 2, and model 3 yielded an accuracy of 88.3%, 85.8%, 86.4%, and 84.6%, a sensitivity of 85.4%, 84.2%, 76.8%, and 78.0%, and a specificity of 91.2%, 87.5%, 96.2%, and 91.2% in the training set and an accuracy of 88.4%, 84.1%, 82.6%, and 82.6%, a sensitivity of 88.6%, 77.1%, 74.3%, and 85.7%, and a specificity of 88.2%, 91.2%, 91.2%, and 79.4% in the validation set, respectively. There was a significant difference between model 4 and model 1 in discriminating the risk rating in gastrointestinal stromal tumors in the training data set (Delong test, *p* < 0.05). The radiomic models based on clinical features and triple-phase CT images manifested excellent accuracy for the discrimination of risk rating of GISTs.

## Introduction

Gastrointestinal stromal tumors (GISTs) are a common type of mesenchymal neoplasm of the gastrointestinal tract that arise from Cajal cells, accounting for 1%–3% of all gastrointestinal malignancies (1, 2). They occur throughout the gastrointestinal tract, most commonly in the stomach (60%–70%), small intestine (20%–25%), followed by duodenum, rectum, colon, and esophagus (3). Generally, about 18%, 35%, and 47% of these tumors were considered benign, malignant potential, and undetermined potential, respectively (4). GISTs are divided into very-low-, low-, intermediate-, and high-risk groups based on the reference guide for prognosis defined by the 2008 National Institutes of Health (NIH) criteria (5, 6). Accurate rating of the risk of GISTs plays a vital role in the decision-making of treatment and outcome (7, 8). The postoperative metastasis and recurrence rates range from 2% to 80% in different risk rating of GISTs, which mainly depend on tumor size, location, and mitotic count (9, 10). With early precise diagnosis, the outcomes and prediction of high-risk GISTs could be improved due to targeted therapy (3, 11).

Currently, abdominal enhanced CT scan is a useful approach in the pre-operative evaluation of GISTs by providing valuable information in relation to the location, size, and blood supply of the tumor and may have potential to predict malignancy risk (3, 12–14). However, these CT features’ assessment is subjective and biased, which is susceptible to observer variability. Radiomics based on CT images is a more quantitative and objective approach to quantify potential biological features of tumor by extracting enormous quantitative characteristics based on tumor’s shape, intensity, size, and texture (15, 16). The predictive value of radiomics based on CT images for predicting the malignancy in GISTs has been reported in previous reports (17–19). Nevertheless, these studies demonstrated excellent predictive performance for risk rating of GISTs using either nonenhanced or enhanced CT images (20–22). So far, to our knowledge, whether the radiomics based on triple-phase CT images combined with clinical features is more preferable for predicting the malignancy in GISTs compared with radiomics based on single-phase CT images has not been reported. Hence, in this study, we aimed to build and validate radiomics models based on triple-phase CT images combining with clinical features for GISTs risk stratification.

## Materials and Methods

### Patients

**Figure 1:**
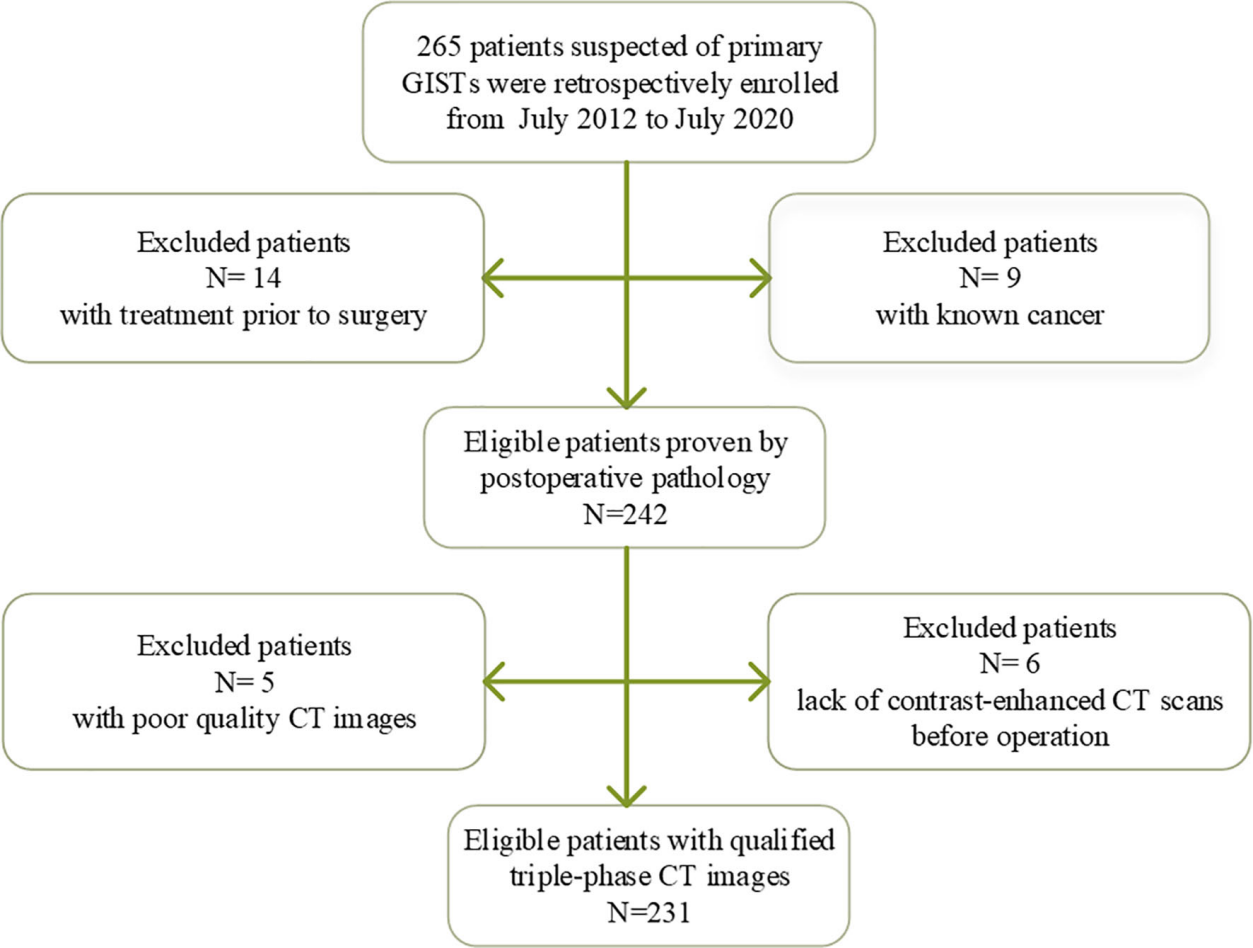
The inclusion and exclusion criteria of patients.

This study acquired the approval of the institutional ethics review board of our hospital; written informed consent was waived due to the retrospective nature of the study. Initially, a total of 265 patients clinically suspected of primary GISTs were recruited in a local hospital from July 2012 to July 2020. The inclusion criteria were as follows: (1) the diagnosis of GISTs was confirmed postoperative pathology; (2) the patients finished contrast-enhanced CT scans within 15 days before operation; (3) clinicopathologic data were integrated; and (4) no treatment prior to surgery. The exclusion criteria included patients with a previous history of GISIs or known cancer or tumor size < 1.0 cm or with poor CT image quality, which may affect target lesion segmentation. Finally, there were 231 patients included in this study. Clinical data were scrutinized and included age, sex, and symptoms (hematemesis or black stool, abdominal pain, or discomfort). The details of inclusion and exclusion criteria are displayed in **Figure 1**.

### CT Examinations and Features

All CT scans including noncontrast CT and contrast-enhanced CT examinations were completed using one of the three CT scanners (SOMATOM Emotion16, SIEMENS, Germany; Definition AS, SIEMENS, Germany; Optima CT680, GE, USA). For contrast-enhanced CT scanning, a total amount of 80–120 ml of contrast medium was injected intravenously at a flow rate of 3–4 ml/s by an automatic triggering injector according to the patient’s weight. After a fast of at least 4 h, all subjects were asked to intake 500–1000 ml of water over 15 min preceding CT scanning. The arterial phase and portal venous phase images were obtained when delaying 25–30 s and 50–70 s after the injection. The parameters of CT scanning were as follows: tube voltage 120–130 kV; tube current 210 mA; slice thickness 1.5 mm; algorithm standard. CT features were analyzed as follows: CT value of unenhanced (CTU), CT value of arterial phase (CTA), CT value of venous phase (CTV), long diameter (LD), short diameter (SD), location, contour, growth pattern (endophytic, exophytic, and mixed), necrosis, calcification, surface ulceration, and intratumoral vessel. Necrosis was defined as unenhanced portion with density ranging from −20 HU to 20 HU, and the presence of calcification with the density above 120 HU. Surface ulceration was described as the endoluminal surface of the lesion showing a focal tissue defect (23). Furthermore, the longest diameter and shortest diameter of the lesion, where it appeared largest and shortest on axial images, were measured, respectively. The CT image review was retrospectively performed by two skilled radiologists (JW and ZN) who were blinded to the clinicopathologic data of all the subjects. Disagreements were solved by consensus.

### Reference Standard and Data Partitioning

According to the NIH criteria, GISTs were divided into very-low-, low-, intermediate-, and high-risk groups based on the tumor size, mitotic count, and tumor site. Furthermore, the study population was classified into two risk grades, varying from a low-malignant (very low and low risk) group to a high-malignant (intermediate and high risk) group.

The reference standard was the pathology based on resection specimens. The total study population was randomly classified into a training data set (80 patients in the low-malignant group, 82 patients in the high-malignant group) and a validation data set (34 patients in the low-malignant group, 35 patients in the high-malignant group) with a ratio of 7:3 according to a computer-generated seed.

### Image Processing and Analysis

**Figure 2:**
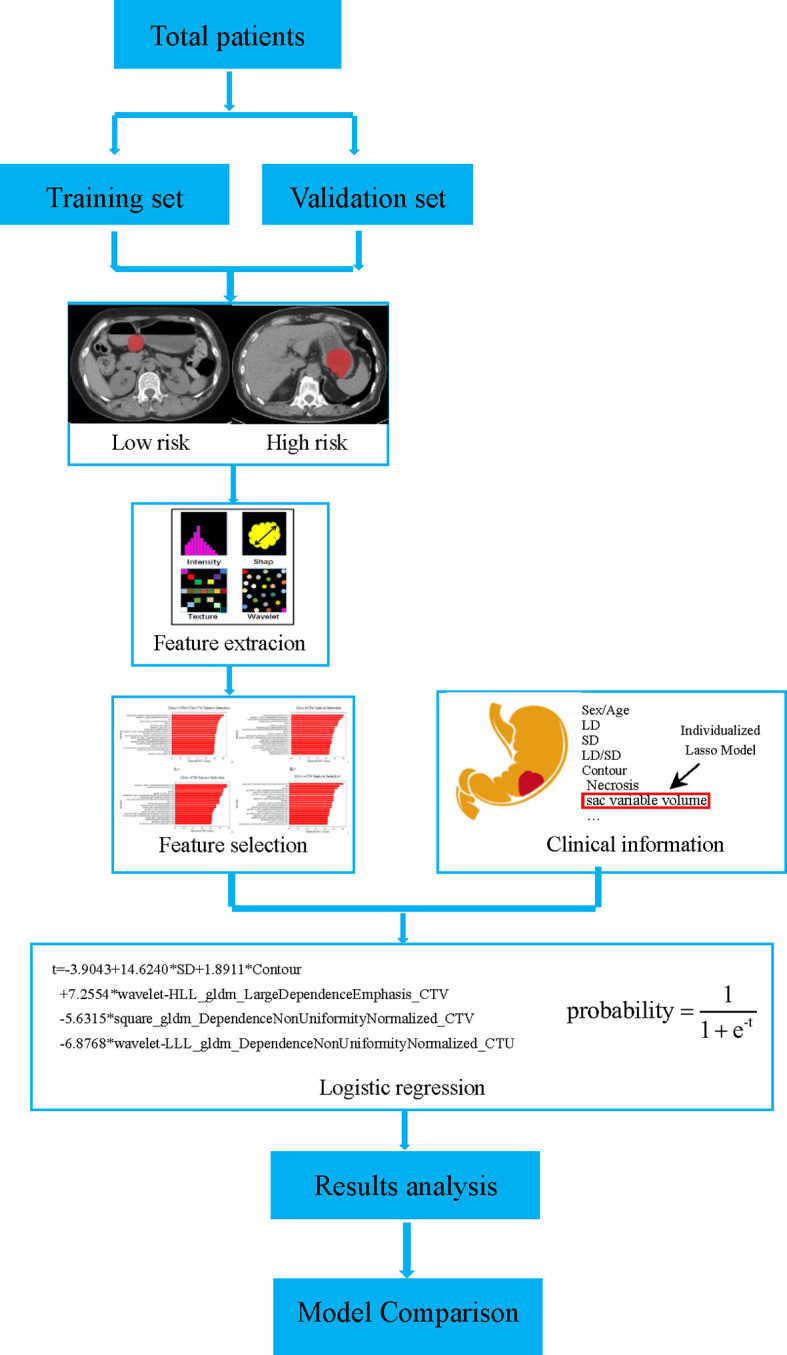
Flow chart of the proposed workflow. GISTs were divided into a training set and a validation set. According to the NIH criteria, the lesion segmentation and features extraction were performed. The radiomics models were built based on CT images combining clinical information, and a comparison between models was also performed.

All the triple-phase CT images were determined using the homogenization process, including (1) data integration, (2) data washing (hiding patient information), (3) data standardization (denoising, unifying window width and window level), (4) data normalization, and (5) data label after structuring. The tumor segmentation was finished by two skilled radiologists, who had 15 (JW) and 15 (ZN) years of experience in abdominal imaging diagnosis by employing the ITK-SNAP software (open source, www.itk-snap.org). All the triple-phase CT images completed the tumor segmentation by the two experienced radiologists who were blinded to GIST risk rating before segmentation. Discrepancies between observers were solved by consensus. In this work, we adopted three-dimensional (3D) segmentation of the region of interest (ROI) that was obtained by overlaying all the single two-dimensional (2D) slices from the ROI with the largest tumor area for each lesion, which was finished by the above two radiologists. Quantitative radiomics features were extracted automatically by employing the software called PyRadiomics (http://www.radiomics.io/pyradiomics. html), as previously described (24, 25). After normalizing these features using Min-Max Normalization method, the Pearson correlation coefficient (PCC) was calculated between each pair of features in order to remove the highly inter-correlated radiomics features (26). If the absolute correlation coefficient (*r*) between each pair features was ≥ 0.8, the feature with the largest mean absolute correlation was excluded (26). Finally, 18 features with the largest PCC were selected to build the stepwise logistic regression models. All models were built with the training data set and were validated on the validation data set. **Figure 2** demonstrates our workflow.

### Statistical Analysis

The statistical analyses were performed using R software, (version 3.6.3; http://www.Rproject.org). A two-sample *t*-test and chi-square test were performed to compare continuous variables and qualitative variables, respectively. The prediction performance of models was assessed on both the training data set and validation data set, with the area under curve (AUC), sensitivity, specificity, and accuracy calculated using the “caret” package (19). Delong test was performed to compare the ROC curve of the models constructed in different data sets. Continuous variables were displayed as mean ± standard deviation (mean ± sd).

## Results

### Clinical Characteristics and CT Features

The details of the clinical and demographic characteristics of GISTs are summarized and compared in **Table 1**. A total of 231 GISTs consisting of 114 low-malignant and 117 high-malignant potential were recruited for this study. No significant differences were found in age, sex, CTU, CTA, CTV, location, calcification, and symptom of tumor between the low-malignant and high-malignant potential groups in either the training data set or the validation data set (with all *p* > 0.05). The LD, SD, contour, necrosis, surface ulceration, and intratumoral vessel between the high-malignant group and the low-malignant group were significantly different in both the training data set and validation data set (all *p* < 0.05). The growth pattern and symptoms between the high-malignant group and the low-malignant group were significantly different in the training data set (with both *p* < 0.05), while not significantly different in the validation data set (with both *p* > 0.05).

**Table 1:** The clinical characteristics and CT features in the training and validation sets.

	Training set			Validation set		
	Low risk *N* = 80	High risk *N* = 82	*p*	Low risk *N* = 34	High risk *N* = 35	*p*
Age	59.14 ± 9.92	58.71 ± 12.84	0.812	61.24 ± 8.41	60.06 ± 10.68	0.613
Sex (Female/Male)	39/41	46/36	0.350	16/18	18/17	0.717
CTU	33.55 ± 9.53	33.55 ± 5.79	1.000	33.78 ± 6.94	35.05 ± 6.21	0.426
CTA	53.00 ± 13.84	56.74 ± 15.07	0.107	53.77 ± 10.90	54.43 ± 13.45	0.823
CTV	66.50 ± 17.41	71.60 ± 18.66	0.079	70.07 ± 15.13	70.24 ± 16.82	0.965
LD (mm)	25.59 ± 10.87	64.24 ± 40.69	**0.000**	24.24 ± 10.70	59.06 ± 34.09	**0.000**
SD (mm)	21.29 ± 9.42	49.45 ± 27.56	**0.000**	20.24 ± 9.60	45.34 ± 20.22	**0.000**
Location			0.420			0.812
Cardia	4	2		2	1	
Fundus	28	22		12	11	
Body	38	49		16	20	
Antrum	10	9		4	3	
Contour			**<0.001**			**<0.001**
Round	42	11		16	7	
Oval	26	15		14	4	
Irregular	12	56		4	24	
Growth pattern			**<0.001**			0.085
Endophytic	44	18		14	9	
Exophytic	26	39		18	18	
Mixed	10	25		2	8	
Necrosis	17	58	**<0.001**	4	24	**<0.001**
Calcification	13	17	0.464	2	7	0.167
Surface ulceration	8	31	**<0.001**	1	11	**0.002**
Intratumoral vessel	1	21	**<0.001**	0	6	**0.036**
Symptom			**0.035**			0.194
Hematemesis or black stool	46	31		20	13	
Abdominal pain or discomfort	11	20		3	5	

p-values written in bold manifest a significant difference between the groups.

CTU, CT value of unenhanced; CTA, CT value of arterial phase; CTV, CT value of venous phase; LD, long diameter; SD, short diameter.

### Results of Radiomics Signature Model

After dimension reduction, 18 of the 1,243 features that remained were used to evaluate whether the radiomics model could distinguish between high-malignant and low-malignant GISTs. The significant radiomics features include three form factor features (SD, LD, and contour), three gray-level co-occurrence matrices (GLCM), three gray-level run length matrices (GLRLM), seven gray-level dependence matrices (GLDM), and two gray-level size zone matrices (GLSZM). **Table 2** presents significant features and coefficients of the four models.

**Table 2:** The significant features and coefficients in the four models.

	Model 4	Model 1	Model 2	Model 3
Intercept	−3.90	−4.12	5.45	−1.45
SD	14.62			9.76
Contour	1.89	2.43	1.39	1.16
wavelet-HLL_gldm_Large Dependence Emphasis_CTV	7.26			
square_gldm_Dependence Non Uniformity Normalized_CTV	−5.63			
wavelet-LLL_gldm_Dependence Non Uniformity Normalized_CTU	−6.88			
wavelet-LLL_gldm_Large Dependence Emphasis_CTU		7.42		
LD			16.22	
wavelet-HLH_glrlm_Long Run Emphasis_CTA			5.22	
wavelet-LHH_glrlm_Run Variance_CTA			−5.59	
wavelet-LLH_glcm_Idmn_CTA			−5.51	
wavelet-LLH_gldm_Small Dependence Low Gray Level Emphasis_CTA			−8.59	
wavelet-HHH_glszm_Small Area Emphasis_CTV				2.19
wavelet-LLL_glcm_Idn_CTV				−5.50
wavelet-LLL_gldm_Large Dependence Emphasis_CTV				−1.05
wavelet-LLL_glrlm_Run Percentage_CTV				−6.29
wavelet-LL_glszm_ZoneEntropy_CTV				2.64
square_gldm_Dependence Non Uniformity Normalized_CTV				0.94
Square root_glcm_Idn_CTV				4.37

SD, short diameter; GLDM, gray-level dependence matrix; LD, long diameter; GLRLM, gray-level run length matrix; GLCM, gray-level co-occurrence matrix; GLSZM, gray-level size zone matrix; Model 4, Clinic + CTU + CTA + CTV; Model 1, Clinic + CTU; Model 2, Clinic + CTA; Model 3, Clinic + CTV; CTU, CT value of unenhanced; CTA, CT value of arterial phase; CTV, CT value of venous phase.

### Diagnostic Efficacy of Four Models

**Figure 3:**
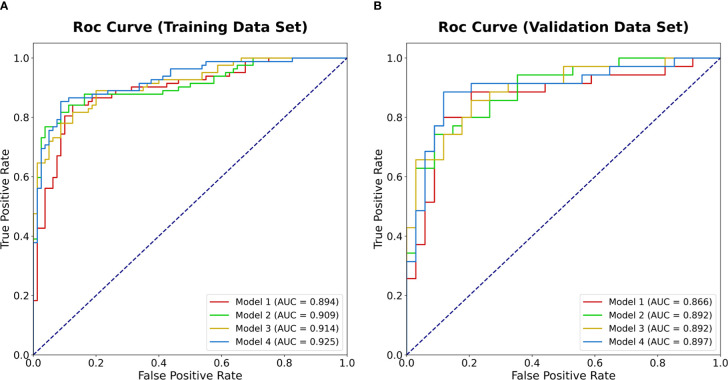
The ROC curves of the four models in predicting malignancy potential of GISTs in the training data set **(A)** and the validation data set **(B)**. AUC, area under the receiver operating characteristic curve; GIST, gastrointestinal stromal tumors.

**Figure 4:**
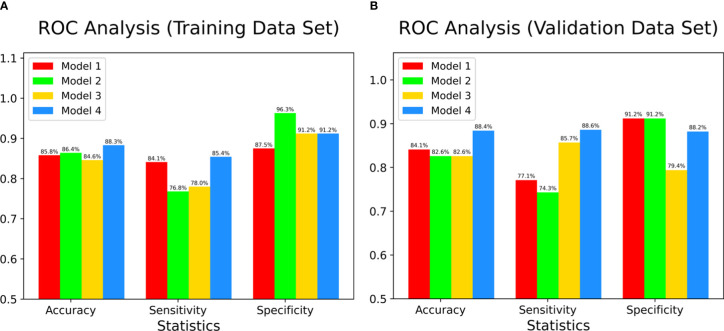
The accuracy, sensitivity, and specificity of four models in both training data set **(A)** and validation data set **(B)**. Color bars indicate radiomics models.

The results of ROC analyses showed that areas under ROC curves (AUC) of model 4 (Clinic + CTU + CTA + CTV), model 1 (Clinic + CTU), model 2 (Clinic + CTA), and model 3 (Clinic + CTV) were 0.925, 0.894, 0.909, and 0.914 in the training set and 0.897, 0.866, 0,892, and 0.892 in the validation set, respectively (**Figure 3**). Model 4, model 1, model 2, and model 3 yielded an accuracy of 88.3%, 85.8%, 86.4%, and 84.6%, a sensitivity of 85.4%, 84.2%, 76.8%, and 78.0%, and a specificity of 91.2%, 87.5%, 96.2%, and 91.2% in the training set and an accuracy of 88.4%, 84.1%, 82.6%, and 82.6%, a sensitivity of 88.6%, 77.1%, 74.3%, and 85.7%, and a specificity of 88.2%, 91.2%, 91.2%, and 79.4% in the validation set, respectively (**Figure 4** and **Table 3**).

**Table 3:** Diagnostic efficacy of four models in the discrimination between low-risk and high-risk GISTs in both training and validation sets.

	Model 4				Model 1				Model 2				Model 3			
	Training set		Validation set		Training set		Validation set		Training set		Validation set		Training set		Validation set	
	Low *N* = 85	High *N* = 77	Low *N* = 34	High *N* = 35	Low *N* = 83	High *N* = 79	Low *N* = 39	High *N* = 30	Low *N* = 96	High *N* = 66	Low *N* = 40	High *N* = 29	Low *N* = 91	High *N* = 71	Low *N* = 32	High *N* = 37
Accuracy	88.3		88.4		85.8		84.1		86.4		82.6		84.6		82.6	
Sensitivity	85.4		88.6		84.2		77.1		76.8		74.3		78.0		85.7	
Specificity	91.2		88.2		87.5		91.2		96.2		91.2		91.2		79.4	
AUC	0.925		0.897		0.894		0.866		0.909		0.892		0.914		0.892	

AUC, area under the receiver operating characteristic curve; Model 4, Clinic + CTU + CTA + CTV; Model 1, Clinic + CTU; Model 2, Clinic + CTA; Model 3, Clinic + CTV; CTU, CT value of unenhanced; CTA, CT value of arterial phase; CTV, CT value of venous phase.

Delong test was also performed on both training and validation sets, and the results showed that there was significant difference between model 4 and model 1 in discriminating the risk rating in gastrointestinal stromal tumors in the training data set (*p* = 0.033). There were no significant differences between the comparison of other models on both training and validation sets, indicating that the models were not overfitting (all *p* > 0.05) (**Table 4**).

**Table 4:** The DeLong test results of the four models.

Cohort	Model 1	Model 2	AUC of Model A	AUC of Model B	*p*-value
Training	Model 4	Model 1	0.925	0.894	0.033
	Model 4	Model 2	0.925	0.909	0.280
	Model 4	Model 3	0.925	0.914	0.308
	Model 1	Model 2	0.894	0.909	0.374
	Model 1	Model 3	0.894	0.914	0.190
	Model 2	Model 3	0.909	0.914	0.704
Validation	Model 4	Model 1	0.897	0.866	0.104
	Model 4	Model 2	0.897	0.892	0.878
	Model 4	Model 3	0.897	0.892	0.826
	Model 1	Model 2	0.866	0.892	0.422
	Model 1	Model 3	0.866	0.892	0.319
	Model 2	Model 3	0.892	0.892	1.000

AUC, area under the receiver operating characteristic curve; Model 4, Clinic + CTU + CTA + CTV; Model 1, Clinic + CTU; Model 2, Clinic + CTA; Model 3, Clinic + CTV; CTU, CT value of unenhanced; CTA, CT value of arterial phase; CTV, CT value of venous phase.

## Discussion

In this retrospective study, we focused on establishing and validating four radiomics models based on triple-phase CT images combining clinical features for distinguishing between low-malignant and high-malignant potential GISTs, which showed satisfactory discrimination. Our results confirmed the forecasting ability of radiomic models based on triple-phase CT images for malignant potential of GISTs, and it may be a potentially useful approach for guiding clinical remedy decision-making before operation in a noninvasive way.

In clinical practice, relative symptoms and subjective CT features could assist in predicting the risk of GISTs for operators intuitively. Subjective CT features such as tumor size, location, contour, hemorrhage, and necrosis could be used to evaluate the risk of GISTs (3, 7). In our study, tumor size (LD and SD), contour, necrosis, surface ulceration, and intratumoral vessel between the high-malignant group and the low-malignant group were significantly different in both the training data set and the validation data set. Recent research showed that prediction nomogram consisting of size, cystoid variation, and mean value had an excellent discrimination in both training and validation sets in GIST patients (19). Our results were partly inconsistent with previous reports, which may result from different clinical settings such as CT scanners, systems, and parameters (27). These discrepancies between subjective CT features indicated that results were limited by reproducibility, which was largely up to subjective experience. GISTs were divided into four risk ratings based on lesion size, location, and mitotic of pathology, which was closely associated with the choice of therapy (10, 28). Previous studies suggested that GISTs smaller than 2 cm could be excised or supervised by endoscopy, while patients with a larger size should undergo complete surgical resection to prevent metastasis or postoperative recurrence (29, 30). However, small GISTs may have aggressive features and remained with a poor outcome, indicating that tumor size was not sufficient in evaluating the malignancy of GISTs due to the complexity of their biological behavior (31). Besides, previous studies have reported that GISTs with hematemesis or sized ≥ 5 cm tend to undergo recurrence, suggesting a poor outcome (32, 33). Hence, a more useful quantitative evaluation approach was required to predict the risk of GIST recurrence especially for those small tumors exhibiting high malignancy. In our study, both tumor size (LD and SD) and contour were significantly different between the low-malignant and high-malignant potential GISTs in both the training data set and the validation data set. Consequently, significant features including tumor size and contour were selected to establish radiomics models, which could work as a component for GISTs’ risk rating.

The repeatability of traditional image analysis was not stable, which was influenced by subjective factors and professional levels. Therefore, an objective and quantitative radiomics approach emerged. Radiomics has offered a novel approach to exploit information encompassed in medical images, which could extract numerous quantitative features from images and have exhibited to improve the preoperative prediction of high-malignant potential GISTs compared with the conventional imaging evaluation methods (7, 16, 19, 34). Previous studies have demonstrated the predictive ability of radiomics features obtained from contrast-enhanced CT for the discrimination of risk rating of GISTs. Ren et al. developed a prediction nomogram using standard contrast-enhanced CT images to discriminate low- from high-malignant potential GISTs with an AUC of 0.935 and 0.933 in the training set and validation set, respectively (19). The radiomics signature of Zhang et al. demonstrated considerable results for the risk stratification of GISTs with an AUC of 0.809 for the validation cohort with contrast-enhanced CT examination (6). The results of Zhang et al. showed that non-contract CT-based radiomics demonstrated equivalent prediction potency for the diagnosis of high-risk GISTs compared to contrast CT-based radiomics (22). None of the studies, however, analyzed whether the radiomics based on triple-phase CT images combined with clinical features is superior for the prediction of the malignant risk of GISTs. Here, we established and validated four models based on triple-phase CT images combining CT features and compared the ROC curves of these models using Delong test. After dimension reduction, 18 of the 1,243 features were selected to establish radiomics models that served as different characteristics of lesions. The significant radiomics features included three form factor features (SD, LD, and contour), three GLCMs, three GLRLMs, seven GLDMs, and two GLSZMs. Among these significant radiomics features, there were two, six, and nine parameters from the plain, arterial phase, and venous phase, respectively. Hence, we speculated that radiomics based on CT images from the arterial phase and venous phase could provide much more information than plain in the discrimination of risk rating in GISTs. The GLCM represents how combinations of discretized intensities of neighboring pixels, or voxels in a 3D volume, are distributed along one of the image directions. Like the GLCM, GLRLM also means the distribution of discretized gray levels in an image or in a stack of images. The GLDM was defined as an alternative to the GLCM, which aimed to capture the coarseness of the overall texture and was rotationally invariant. The GLSZM counts the number of groups (or zones) of linked voxels (35, 36). Briefly, these significant features represented lesions’ internal heterogeneity of morphology, density, texture, and distribution. Heterogeneity was an accepted characteristic of malignant tumors and believed to be positively relative to the degree of tumor malignancy, which had vital clinical significance (37). The results of the Delong test showed that there was a significant difference between model 4 and model 1 in discriminating the risk rating in gastrointestinal stromal tumors in the training data set (*p* < 0.05), whereas this difference was not confirmed in the validation data set, which indicated considerable predictive potential in GISTs. The results of Zhang et al. demonstrated that the radiomics signature from nonenhanced CT (NE-RS) had a high AUC of 0.967 and 0.941 on the internal validation cohort and the external validation cohort for GIST malignancy prediction, respectively (22). Our NE-RS had a lower AUC (<0.9) compared to this study in both training and validation data sets. The underlying reason for this discrepancy might be due to difference in different clinical settings such as different scanners or parameters. Moreover, the similarity was that these studies adopted portal phase CT images to establish and validate radiomics signature. In short, our results confirmed that NE-RS had admirable discriminating ability for the prediction of the malignancy potential of GISTs, which may have important clinical instructive significance since nonenhanced CT was more conveniently applied for the preoperative diagnosis in GISTs.

Remarkably, radiomics features in this work were extracted from 3D images of whole lesion rather than 2D images with the largest tumor area. Previous studies have confirmed that analyses using 3D images could supply more ample information about the lesion than 2D images since all the applicable slices were taken into consideration, which may enhance the accuracy of discrimination (17, 38, 39). Hence, radiomics based on 3D images may more precisely reveal the heterogeneity of lesion for examining GISTs compared with 2D images.

However, several limitations still existed that should not be ignored in this present work. First, all data were obtained from a single center, and a multicenter study needs to be designed for further evaluation and validation. Second, selective bias could not be completely avoided due to the retrospective nature of our study design. Nevertheless, all the patients were consecutively included. Third, all CT scans including noncontrast CT and enhanced CT scanning were completed with different CT scanners, which may lead to some possible confounding factors. Finally, we did not compare the algorithms of feature selection and extraction, which could also influence the model performance.

## Conclusion

In conclusion, we hereby concluded that radiomic models based on clinical features and triple-phase CT images manifested satisfactory performance for the discrimination of low- and high-malignant potential GISTs. We stress the potentiality of radiomics analysis based on clinical features and triple-phase CT images as a noninvasive technique to achieve an accurate diagnosis ahead of surgery.

## Data Availability Statement

The raw data supporting the conclusions of this article will be made available by the authors, without undue reservation.

## Ethics Statement

The studies involving human participants were reviewed and approved by the Medical ethics committee of Zhejiang Academy of traditional Chinese medicine. The ethics committee waived the requirement of written informed consent for participation. Written informed consent was obtained from the individual(s) for the publication of any potentially identifiable images or data included in this article.

## Author Contributions

MS, JW, GC, ZN, LH, ZF, JH, and ZX conceived and designed the study. MS, JW, GC, and LH contributed to the literature search. JW, ZN, JH, and ZX contributed to data collection. MS, JW, GC, LH, and ZN contributed to data analysis. MS, GC, ZN, JH, LH, and JW contributed to data interpretation. MS, JW, ZN, and JW contributed to the figures. MS, GC, and JW contributed to writing of the report. All authors contributed to the article and approved the submitted version.

## Conflict of Interest

Authors LH, ZF and GC were employed by Hangzhou Jianpei Technology Company.

The remaining authors declare that the research was conducted in the absence of any commercial or financial relationships that could be construed as a potential conflict of interest.

## Publisher’s Note

All claims expressed in this article are solely those of the authors and do not necessarily represent those of their affiliated organizations, or those of the publisher, the editors and the reviewers. Any product that may be evaluated in this article, or claim that may be made by its manufacturer, is not guaranteed or endorsed by the publisher.
